# NRas activity is regulated by dynamic interactions with nanoscale signaling clusters at the plasma membrane

**DOI:** 10.1016/j.isci.2022.105282

**Published:** 2022-10-09

**Authors:** Oren Yakovian, Julia Sajman, Michal Alon, Rand Arafeh, Yardena Samuels, Eilon Sherman

**Affiliations:** 1 Racah Institute of Physics, The Hebrew University, Jerusalem 91904, Israel; 2 Department of Molecular Cell Biology, Weizmann Institute of Science, 7610001 Rehovot, Israel; 3 Department of Molecular Oncology, Dana Farber Cancer Institute, Boston, MA, USA

**Keywords:** Biological sciences, Cell biology, Biophysics, Molecular biology

## Abstract

NRas is a key mediator of the mitogenic pathway in normal cells and in cancer cells. Its dynamics and nanoscale organization at the plasma membrane (PM) facilitate its signaling. Here, we used two-color photoactivated localization microscopy to resolve the organization of individual NRas and associated signaling proteins in live melanoma cells, with resolution down to ∼20 nm. Upon EGF activation, a fraction of NRas and BRAF (dis)assembled synchronously at the PM in co-clusters. NRas and BRAF clusters associated with GPI-enriched domains, serving as possible nucleation sites for these clusters. NRas and BRAF association in mutual clusters was reduced by the NRas farnesylation inhibitor lonafarnib, yet enhanced by the BRAF inhibitor vemurafenib. Surprisingly, dispersed NRas molecules associated with the periphery of self-clusters of either Grb2 or NF1. Thus, NRas-mediated signaling, which is critical in health and disease, is regulated by dynamic interactions with functional clusters of BRAF or other related proteins at the PM.

## Highlights

• Two-color PALM resolved the dynamic interactions of NRas in live melanoma cells
• Upon EGF activation, NRas and BRAF (dis)assembled synchronously in co-clusters
• NRas and BRAF co-clustering was reduced by lonafarnib, yet enhanced by vemurafenib
• NRas signaling is regulated by interactions with BRAF, Grb2, NF1, and GPI clusters

## Graphical abstract

**Figure:**
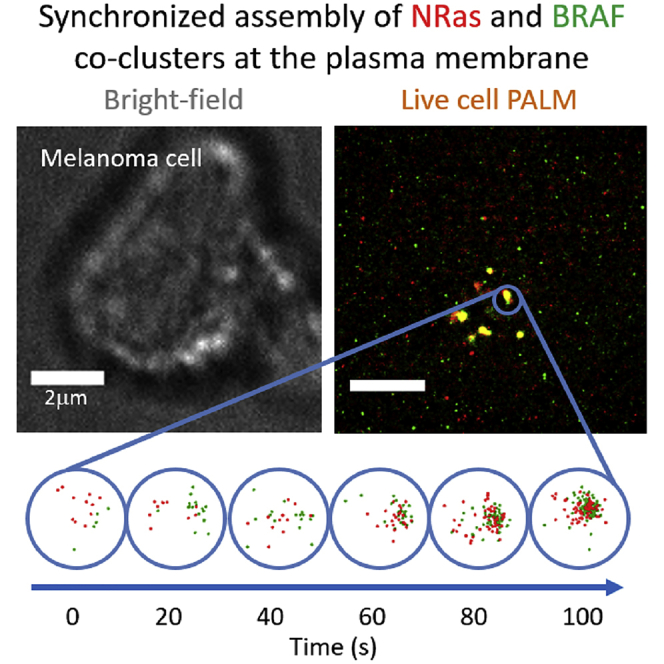

## Introduction

Ras and RAF family proteins are key mediators of the mitogenic pathway. Their signaling drives important cell processes such as proliferation, survival, motility, and differentiation. Because Ras and Raf are common oncogenes (Cargnello and Roux, 2012), the mechanisms of Ras-RAF signaling have been extensively studied in health and in cancer using a wide range of experimental and theoretical techniques (Terrell and Morrison, 2019). The goal is often to study ways to target the aberrant signaling of these oncogenic proteins for effective cancer therapy (Santarpia et al., 2012). Specially, gain-of-function missense mutations in Ras isoforms are frequently activated in human cancer (Schubbert et al., 2007; Hobbs et al., 2016). Still, the direct inhibition of aberrant activity of oncogenic Ras mutants remains highly challenging (Khan et al., 2020; Samatar and Poulikakos, 2014). Thus, mechanistic understanding of signaling downstream Ras may provide novel opportunities for such inhibition, and remains an outstanding goal in research of cell signaling and in cancer (Khan et al., 2020; Samatar and Poulikakos, 2014).

The mitogenic pathway is initiated by membrane receptor-tyrosine kinases binding of epidermal growth factors (EGF) and their phosphorylation (Keshet and Seger, 2010). Grb2 and SOS complexes are recruited to the activated receptors (Schlessinger, 1993). In turn, SOS serves as a guanine exchange factor (GEF) to activate Ras molecules that are recruited to the plasma membrane (PM) (Schlessinger, 1993). The signal of activated Ras then activates RAF family proteins, MEK, and Erk. Activated Erk enters the nucleus where it drives cell growth and proliferation (Morrison, 2012). The activity of Ras is regulated to avoid excess signaling and cell proliferation. NF1 is a main inhibitor that serves as a GTPase-activating protein (GAP) (Marchuk et al., 1991; Bollag et al., 1996), while additional proteins may contribute to the regulation of Ras activity (Arafeh et al., 2015).

Recent studies have used super-resolution microscopy, FRET, and immune-EM to show the membrane recruitment of Ras and RAF (Prior et al., 2003), Ras trafficking (Schmick et al., 2015) and dynamics at the PM (Lee et al., 2019), and the formation of Ras nano-clusters at the PM (Nan et al., 2015; Yakovian et al., 2021; Parkkola et al., 2021). These studies have shown that the nanoscale organization of Ras and its effectors plays a critical role in its signaling (Zhou and Hancock, 2015). More recently, we and others have shown the self- and co-clustering of NRas and BRAF at the PM in fixed cells (Nan et al., 2015; Yakovian et al., 2021; Prior et al., 2003). Still, the dynamics of Ras-RAF nanoscale organization and signaling at the PM remain poorly understood. Moreover, immune-EM suffers from multiple artifacts that critically compromise its ability to resolve the nanoscale organization of proteins at the PM. Such artifacts include poor labeling efficiency, multiple clustering artifacts (Griffiths and Lucocq, 2014), and the requirement to rip the cells from the sample surface before labeling and imaging (Leung et al., 2017). On the other hand, FRET is sensitive to immediate interactions between proteins at short distances (<∼8 nm), but not to higher order complexes and patterns at ∼10–200 nm. Thus, single-molecule localization microscopy can assist in studying the nanoscale organization of Ras and its interactions with upstream and downstream proteins.

Here, we used two-color photoactivated localization microscopy (PALM) (Subach et al., 2009) to resolve the nanoscale organization of NRas and multiple signaling proteins, and their dynamics in live melanoma cells. PALM provided NRas and BRAF localization accuracy of ∼20–30 nm with an effective time resolution of 5 s. Strikingly, upon EGF activation, a fraction of NRas and BRAF assembled in pronounced and dynamic co-clusters at the PM. Assembly and disintegration of NRas and BRAF co-clusters were highly synchronized and took ∼1 min. Clustered NRas molecules associated with GPI-enriched domains, which are implicated as nucleation sites (Eisenberg et al., 2011). Surprisingly, dispersed NRas molecules associated with proximal, yet non-overlapping self-clusters of Grb2 and of NF1 at their periphery. Taken together, our results show that NRas activity is mediated and regulated by its dynamic interactions with functional (Grb2, BRAF, NF1, and GPI-nucleated) clusters at the PM. Thus, our study sheds new light on the dynamics and mechanisms of NRAS signaling at the PM, which is critical in health and disease.

## Results

### NRas and BRAF get dynamically recruited and associate in co-clusters at the plasma membrane of live melanoma cells

**Figure 1:**
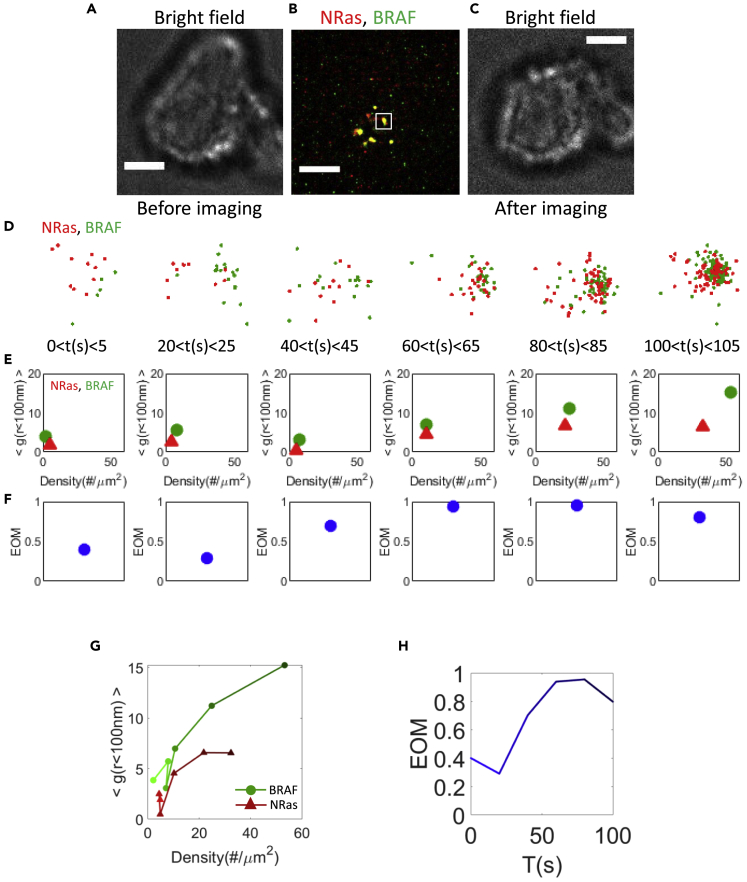
NRas and BRAF form co-clusters at the plasma membrane of live melanoma cell upon EGF activation 108T cells were dropped onto EGF-coated coverslips and imaged upon initial contact between the cells and coverslip. (A) Bright field image of a melanoma cell before coverslip attachment. Shown is a representative cell (N = 15). Bars – 2 μm. (B) Two-color PALM imaging of the cell in (A). Single SMLM frames were reconstructed from 250 camera frames, yielding a temporal sequence of images every 5 s (see STAR methods). Shown are PAmCherry-NRas and PAGFP-BRAF expressed by the cell. (C) Bright field image of the cell in (A), 4 min after its attachment to the coverslip. (D) Dynamics of single BRAF (green) and NRas (red) proteins at the PM in zoomed region of (B). (E) A two dimensional map of self-clustering (value of g(0–100)) vs. protein density. Values are shown for individual cluster as discs, either for BRAF (green) or NRas (red). The positions of discs change with time progress after activation. (F) The extent of mixing (EOM) of BRAF and NRas for a single cluster. (G) The dynamic changes of the proteins self-clustering through the two-dimensional map shown in (E). The color of the line trajectories darken with time, as specified in panel (E). (H) The EOM trajectories of BRAF and NRas for a single cluster shown in (F). This trajectory also darkens with time, as in (G). Estimated errors related to the g(r) and EOM measures in panels (E–H) are shown in Figure S1H.

We used two-color PALM to study the dynamics of NRas and BRAF organization at the PM of live (108T) melanoma cells, in single molecule detail. In these melanoma cells, PAmCherry-NRas and PAGFP-BRAF could both be localized with ∼20–30 nm (Figures S1A and S1B), with an effective time resolution of 5 s (Figures 1 and S2; Video S1).

Video S1. Co-clustering dynamics of NRas and BRAF, related to Figure 1 A movie showing PM recruitment and co-cluster formation (in regions 1–3) or disintegration (in regions 1,2). The movie was captured using two-color PALM live imaging of NRas and BRAF in 108T melanoma cell. Shown at ×4 rate.

Specifically, we were interested in studying the early reorganization of NRas and BRAF upon cell activation by EGF. For that, cells were dropped onto EGF-coated coverslips. The imaging started upon initial contact between the cell and EGF-coated coverslip in total internal reflection (TIRF) mode (Figures 1A, 1B, and 1C). Imaging showed arrival and dynamic co-clustering of NRas and BRAF at the PM (Figures 1B and 1D). To quantify the extent of relative self-clustering for each protein in individual clusters, we defined a two-dimensional map of protein surface density (x axis) and the average values of univariate pair-correlation function (PCF, or g(r)) (y axis) (Yakovian et al., 2021). Values corresponding to individual clusters are shown as colored discs (Figure 1E; green for BRAF and red for NRas). In the shown example, the clustering extent of NRas was up to g_11_ of ∼7 and density of 30 molecules/μm^2^, while BRAF clustering extent was of g_11_∼15 and density of 55 molecules/μm^2^. These values of g_11_ indicate the extent of significant short-range self-clustering of NRas and BRAF over a random (Poisson) distribution (see further details in STAR methods).

To study the mutual colocalization of BRAF and NRas within the clusters, we used a normalized bivariate PCF statistics (BPCF) named “extent of mixing” (EOM; Figure 1F; Nan et al., 2015; Yakovian et al., 2021). In this analysis, an EOM value of 0 means no interaction between the molecules, while a value of 1 indicates homogenous mixing within the clusters (see also STAR methods). We also represented dynamic changes of the proteins self-clustering using a two-dimensional map (Figure 1G, trajectories darken with time), and their co-clustering using EOM (Figure 1H). We use this more compact presentation in the subsequent Figures below. We further note that relatively low counts of molecules involved in clustering (<∼20 molecules/μm^2^ of NRas, BRAF, or both) translate into significant fluctuations in the presented measures of g(r < 100 nm) and EOM (see Figure S1H for presentation of these fluctuations as a function of molecular densities in individual clusters). The high fluctuations in these measures (namely, molecular densities, g(r), and EOM) highlight the stochastic nature of (co)cluster formation. Quantitative estimates of these measures should be best considered at the more stable stages of cluster formation.

### Formation and disintegration dynamics of NRas and BRAF co-clusters occurs within ∼1–2 min

**Figure 2:**
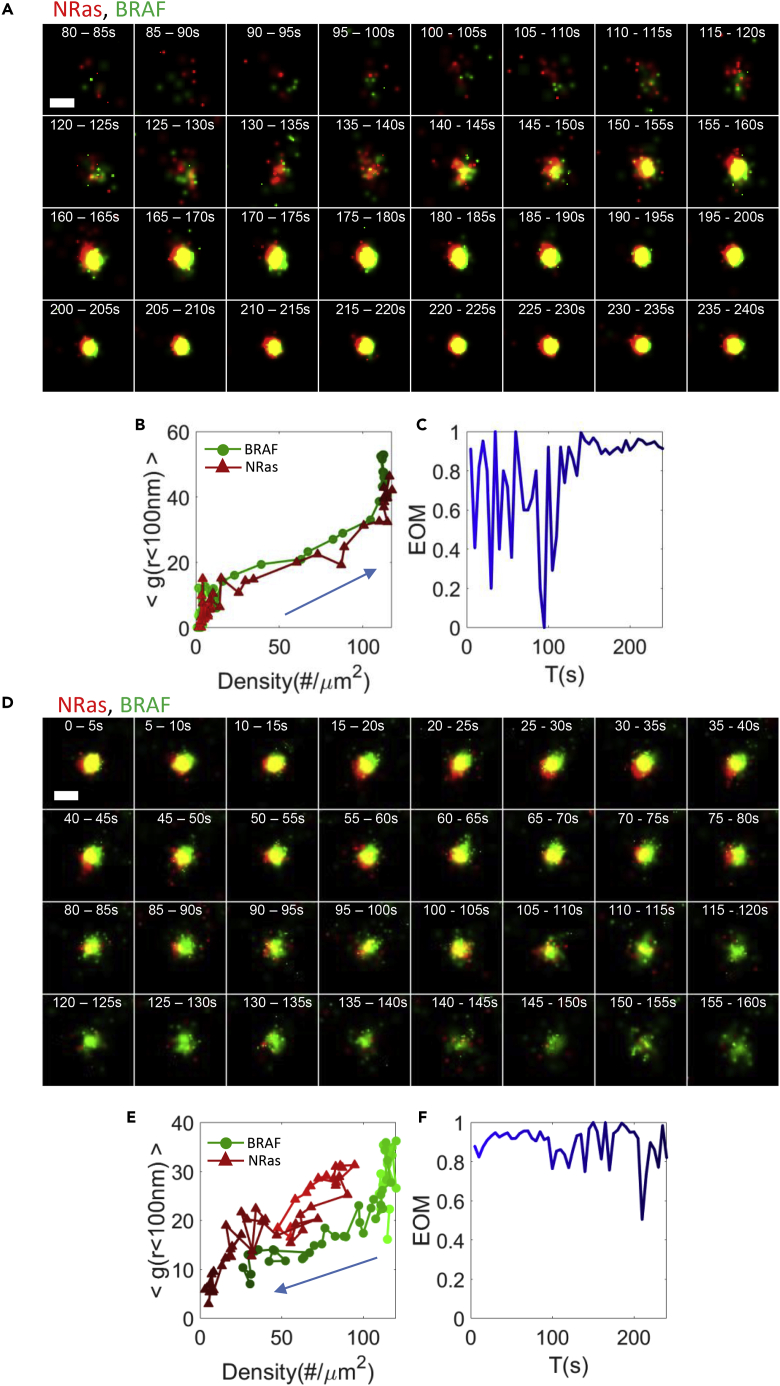
NRas and BRAF coalesce into membrane co-clusters and disperse back (A) Two-color PALM live imaging of co-cluster formation of BRAF (green) and NRas (red) at the plasma membrane of melanoma cells. Effective imaging rate of 5 s/frame. Bar – 200 nm. (B) The dynamic changes of the proteins organization through the two-dimensional density (X axis) and self-clustering (Y axis) map upon co-cluster formation. (C) The EOM trajectories of BRAF and NRas upon co-cluster formation. (D) Two-color PALM live imaging of co-cluster disintegration of BRAF (green) and NRas (red) at the plasma membrane of melanoma cells. Effective imaging rate of 5 s/frame. Bar – 200 nm. (E) The dynamic changes of the proteins organization through the two-dimensional density (X axis) and self-clustering (Y axis) map upon co-cluster disintegration. (F) The EOM trajectories of BRAF and NRas upon co-cluster formation. The color of the line trajectories in panels (B, C, E, and F) darkens with time. Estimated errors related to the g(r) and EOM measures in panels (B, C, E, and F) are shown in Figure S1H.

Next, we focused on specific clusters of NRas and BRAF to study the timescale of NRas and BRAF recruitment to the PM, and their cluster formation (Figure 2A). Most of clusters were created by recruitment of individual proteins to the PM. Cluster formation could be captured by an increase in surface density and the extent of protein self-clustering for both NRas and BRAF (Figure 2B). NRas and BRAF became denser (∼100 proteins per cluster). We note that the number of proteins that are reported here was collected from imaging during 5 s. These numbers should be regarded mostly as relative or as rough estimates of the absolute protein counts (see STAR methods). Strikingly, the two proteins highly mixed together in the forming clusters, until reaching a maximum association in the stably formed co-clusters after ∼60–100 s (Figures 2A and 2C).

We could also trace the nanoscale organization of NRas and BRAF during cluster disintegration. The two-dimensional map indicated that the merged clusters of NRas and BRAF got separated and diffused at the PM. In contrast, internalization of the co-clusters would have likely occurred in a sharper step of co-cluster disappearance. The process of disintegration of BRAF and NRas co-cluster also took ∼100 s and demonstrated reduction in NRas, and then in BRAF (Figures 2D–2F).

### NRas and BRAF co-clustering is correlated and synchronized

**Figure 3:**
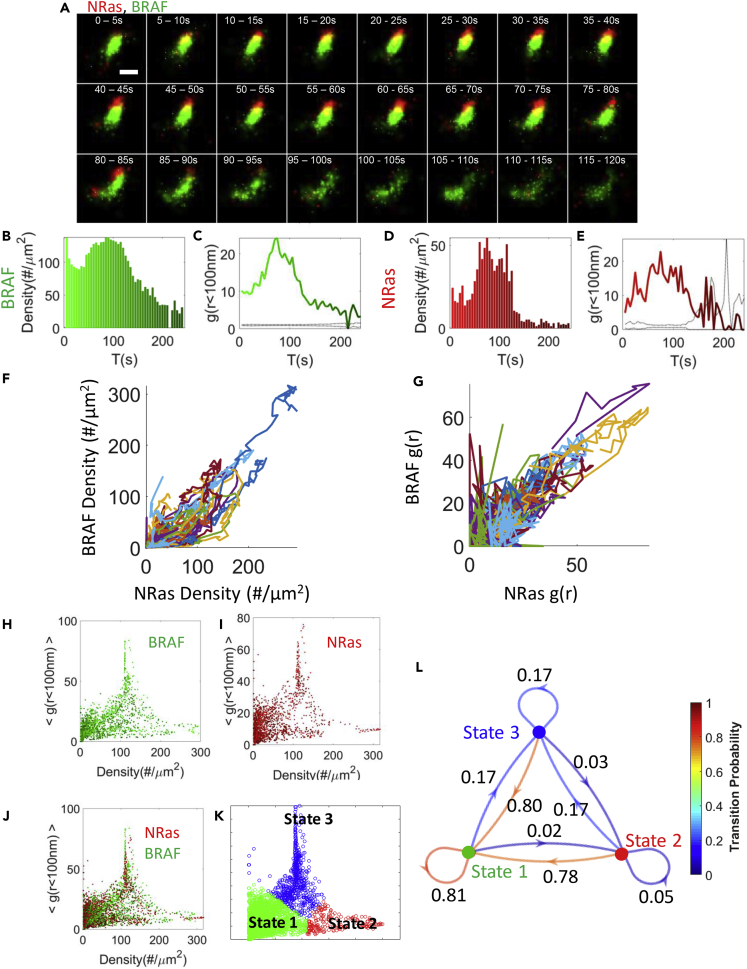
NRas and BRAF co-clustering is correlated and synchronized (A) Two-color PALM live imaging of BRAF (green) and NRas (red) at the plasma membrane of melanoma cell on EGF-coated coverslip. NRas and BRAF were imaged over 120 s. Bar – 200 nm. (B and D) The density over time of BRAF (B) and NRas (D) at the PM. Specified values are for a single co-cluster at an area of 1.5 × 1.5 μm, shown in A (C, E) The pair correlation function (PCF) [value of g(0–100)] over time of BRAF (C) and NRas (E). (F and G) The correlation of density (F) and PCF (G) of BRAF vs. NRas for 41 clusters. (H–J) Classification of NRas and BRAF co-clustering dynamics into 3 states. Organization at the PM in the two-dimensional density vs. self-clustering map overtime of BRAF (H; green discs) and NRas (I; red discs), and their overlap (J). Each point represents the position of a single cluster during 5 s. There are 48 time-points for each of 41 clusters. The color of the points darkens with time. (K) Division of the distribution in (J) into 3 states using Gaussian mixture model (GMM). Monomers or dispersed domains with low molecular density and low extent of self-clustering populate state 1 (green). Molecular domains with high density but low self-clustering populate state 2 (red). Clusters, having high molecular densities and high extent of self-clustering populate state 3 (blue). (L) Markov chain diagram with the transition probability specified between the states. Estimated errors related to the g(r) and EOM measures in panels (C, E, G) are shown in Figure S1H.

It seemed that NRas and BRAF were recruited into clusters and dispersed from them together (Figure 3A). To study these peculiar dynamics, we first plotted the densities of NRas and BRAF over time and side-by-side. We noted that the densities showed similar dynamics (Figures 3B and 3D). We also observed similar dynamics in the extent of self-clustering of these two proteins (Figures 3C and 3E). Strikingly, plotting the time-dependent density of NRas vs. BRAF in 41 clusters showed a high correlation and an average Pearson’s correlation of 0.81 ± 0.03 (Figure 3F). The correlation of the time-dependent self-clustering of NRas and BRAF had a Pearson’s correlation of 0.57 ± 0.06 (Figure 3G).

### Co-clustering dynamics of NRas and BRAF can be captured in 3 states

To better resolve the dynamics of NRas and BRAF (co)clustering, we plotted the coordinates of a large number of individual clusters in our 2-dimensional density-PCF map (Figure 3H for BRAF and Figure 3I for NRas; Figure 3J for both). We noticed that these coordinates could be clustered into 3 distinguishable states (see STAR methods for clustering approach). State 1 (in green, Figures 3J, 3K, and 3L) corresponded to monomers or dispersed domains with relatively low molecular density and low extent of self-clustering. State 2 (in red) corresponded to molecular domains with relatively high density but low self-clustering extent. State 3 (in blue) corresponded to mature clusters, having relatively high molecular densities and high extent of self-clustering.

We could then calculate the transition probability between all states using a Markov chain diagram (Brooks, 1998) (see STAR methods). We found that State 1 (dispersed domains) was the most stable state, followed by state 3—the mature clusters. State 2 (dispersed clusters) was the least occupied, i.e. with the lowest transition probability into this state (Figure 3L). This state might involve a separate formation mechanism than states 1 and 3 that is relatively less frequent or it could be a transient state between states 1 and 3.

### NRas and BRAF clusters associate with GPI-enriched domains

**Figure 4:**
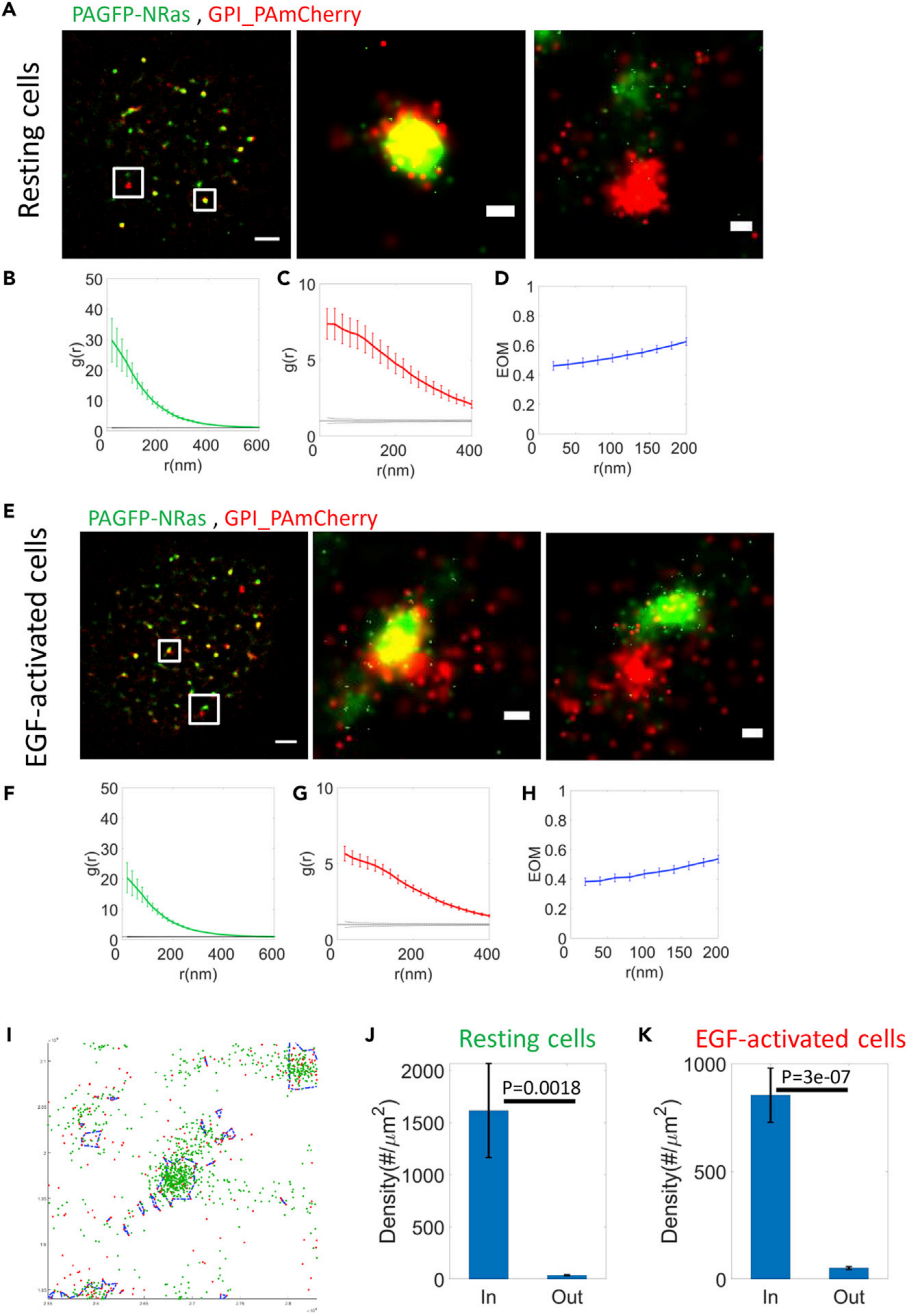
NRas clusters at GPI-enriched domains (A and E) Two-color PALM imaging of resting and EGF-activated 108T melanoma cells expressing PAGFP-NRas and GPI-PAmCherry. Cells were seeded on the coverslip for 2 days before fixation. Shown are representative cells (N = 25 for resting cells, N = 33 for EGF-activated cells). Bars - 2 μm (left) and 200 nm (zoomed images). (B, C, F, and G) PCF of PAGFP-NRas (green) and GPI-PAmCherry (red). (D and H) The EOM of NRas and GPI (averaged over all cells; see analyses for further details). (I) Selected GPI (red points) enriched regions (blue polygons) in a representative area of NRas (green points) and GPI. The area was selected from a two-color PALM image, as in panels A,E. (J and K) The density of NRas in and out of the GPI-enriched domains at the PM of the resting (J) and EGF-activated (K) melanoma cells.

Next, we studied the possible mechanism that allows NRas and BRAF co-clustering at the PM. For that, we imaged by two-color PALM GPI-PAmCherry and PAGFP-NRas in fixed cells. GPI (glycosyl-phosphatidylinositol) is a well-known marker of cholesterol-rich PM domains (lipid raft marker) (Sharma et al., 2004). Both molecules formed pronounced clusters (Figures 4A–4D). We also found that a considerable fraction (21.3 ± 3.7% for resting cells, and 16.1 ± 2.9% for EGF-activated cells), but not all of NRAs and GPI molecules were in the same domains (Figure 4A). We also found similar co-clustering of NRas and GPI at the PM of 108T cells upon EGF activation (Figures 4E–4H, S3G, and S3H).

**Figure 5:**
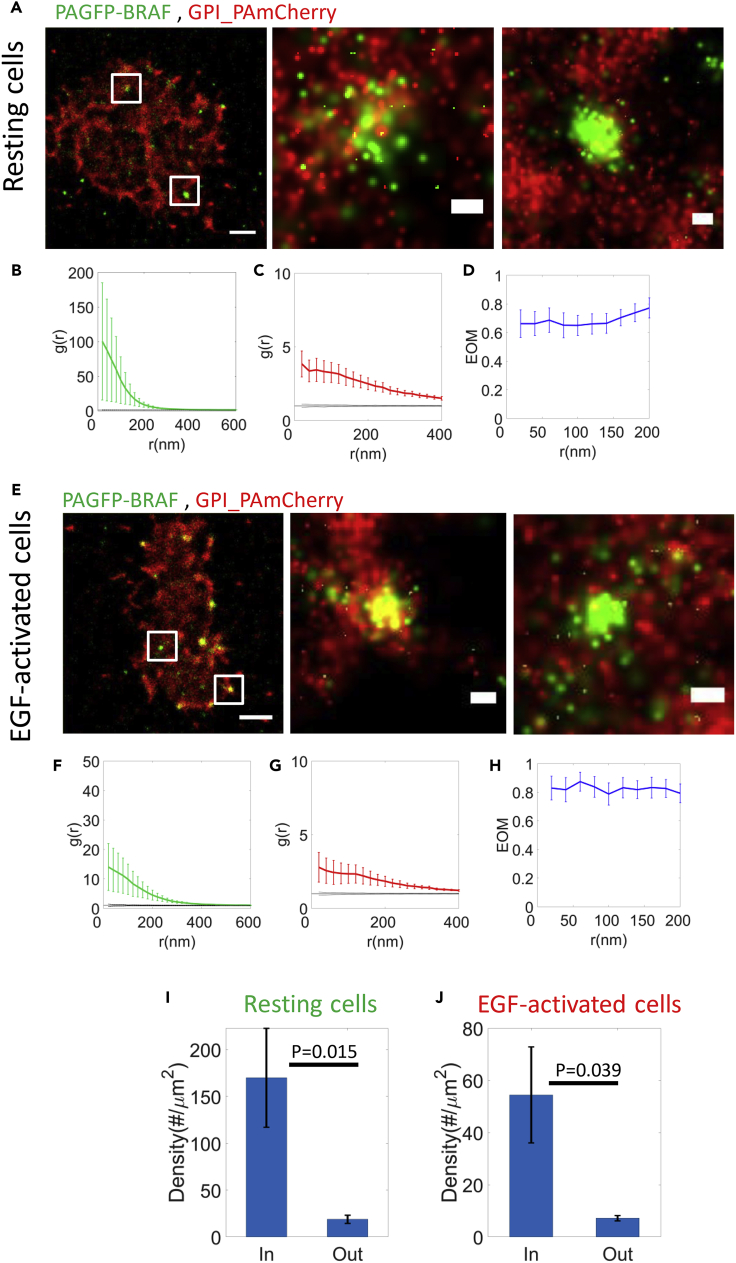
BRAF clusters at GPI-enriched domains (A and E) Two-color PALM imaging of resting and EGF-activated 108T melanoma cells expressing PAGFP-BRAF and GPI-PAmCherry. Cells were seeded on the coverslip for 2 days before fixation. Shown are representative cells (N = 12 for resting cells, N = 13 for EGF-activated cells). Bars - 2 μm (left) and 200 nm (zoomed images). (B, C, F, and G) PCF of PAGFP-BRAF (green) and GPI-PAmCherry (red). (D and H) The EOM of NRas and GPI (averaged over all cells; see analyses for further details). (I and J) The density of BRAF in and out of the GPI-enriched domains at the PM of the resting (I) and EGF-activated (J) melanoma cells.

We defined GPI-enriched domains (see STAR methods) and quantified the density of NRas molecules within these domains vs. their density outside these domains (Figures 4I and S3). The density of NRas within the domains was much (>10-fold) and significantly (p = 0.0018) higher than outside GPI-enriched domains (Figures 5J and 5K). Since GPI is membranal, while NRas undergoes dynamic recruitment to the membrane, our results suggest that GPI-enriched domains can serve as possible recruitment sites for NRas. Indeed, GPI-enriched domains are known as critical region for protein interactions and cell signaling (Simons and Ikonen, 1997). Still, these results do not preclude additional sites for NRas recruitment (e.g. as found for KRas (Zhou et al., 2017)).

Our results so far show the mutual spatial organization of NRas and BRAF and of NRas and GPI. Thus, we next studied the mutual organization of BRAF and GPI at the PM. For that, we performed two-color PALM GPI-PAmCherry and PAGFP-BRAF in fixed melanoma cells. Both molecules formed pronounced clusters (Figures 5A–5D). We also found, as for NRas, a considerable fraction (55.2 ± 10.7% for resting cells, and 61.2 ± 10.3% for EGF-activated cells; but not all) of BRAF and GPI molecules in same domains (Figures 5A and 5E). We also found a similar co-clustering of BRAF and GPI at the PM of 108T cells upon EGF activation (Figures 5E–5H). The density of BRAF within the GPI-enriched domains was much (>7-fold) and significantly (p = 0.015) higher than outside GPI-enriched domains (Figures 5I, 5J, S4A, and S4B).

Taken together, the spatial organization of NRas, BRAF, and GPI at the PM implicates GPI-enriched domains as one of the possible pre-arranged nucleation sites for NRas and BRAF clusters individually, and thus also for the mutual NRas and BRAF clusters that we found.

### NRas and BRAF clusters are affected by inhibitors of NRas farnesylation and BRAF activity

**Figure 6:**
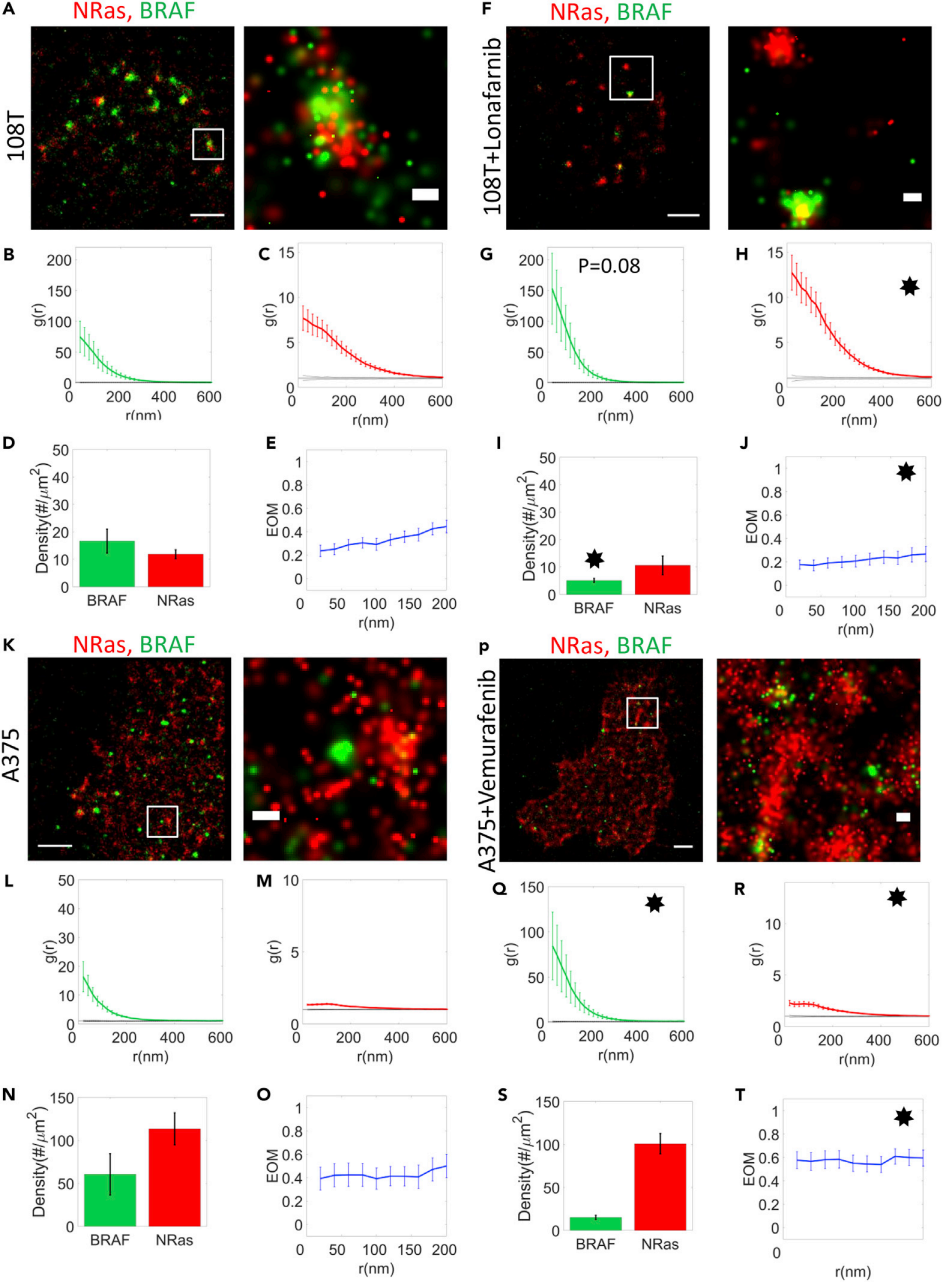
NRas and BRAF clusters are affected by inhibitors of NRas farnesylation and BRAF activity (A and F) Two-color PALM imaging of 108T melanoma cells with and without farnesyltransferase inhibitor (Lonafarnib) expressing PAGFP-BRAF and PAmCherry-NRas. Cells were seeded and treated on the coverslip for 2 days before fixation. Shown are representative cells (N = 18 for untreated cells, N = 17 for treated cells). (B, C, G, and H) PCF of PAGFP-BRAF (green) and PAmCherry-NRas (red). (D and I) The density of BRAF and NRas at the PM. (E and J) The EOM of NRas and BRAF (K and P) Two-color PALM imaging of A375 melanoma cells with and without BRAFi (Vemurafenib) expressing PAGFP-BRAF and PAmCherry-NRas. Cells were seeded and treated on the coverslip for 2 days before fixation. Shown are representative cells (N = 14 for untreated cells, N = 34 for treated cells). (L, M, Q, and R) PCF of PAGFP-BRAF (green) and PAmCherry-NRas (red). (N and S) The density of BRAF and NRas at the PM. (O and T) The EOM of NRas and BRAF (averaged over all cells; see analyses for further details). Stars in treated panels represent a significant difference (p < 0.05) relative to untreated panels. Error bars in all relevant panels are SEM due to measurements on multiple cells. Bars - 2 μm (left) and 200 nm (zoomed images).

We further used targeted drugs to study their potential effect on NRas and BRAF clustering, as well as their association within mutual clusters. Specifically, we used the farnesyl transferase inhibitor lonafarnib to inhibit farnesylation of NRas, and thus its recruitment to the PM in 108T cells (Proietti et al., 2020) (see STAR methods). Treated cells demonstrated elevated self-clustering of BRAF and NRas (Figure 6; compare panels A–C with F–H, respectively), while the BRAF recruitment to the PM was significantly reduced (Figure 6; compare D,I). Also, NRas and BRAF association in mutual clusters was significantly reduced (Figure 6; compare E,J). We propose that the effect of lonafarnib on NRas promoted its self-clustering, yet abrogated its ability to recruit BRAF to these NRas clusters.

We further hypothesized that inhibition of BRAF activity may also be related to its self-clustering and association with NRas in mutual clusters. Vemurafenib is a specific inhibitor of oncogenic BRAF mutants (Namely, BRAF^V600E, V600D, V600R^ (Proietti et al., 2020)) by interfering with their ATP-binding site. Because 108T cells contain only wt BRAF, we studied the effects of vemutrafenib in A375 melanoma cells that express BRAF^V600E^. Notably, NRas clustering in these cells was much lower than in 108T cells, and their PM density was much higher, both regardless of vemurafenib treatment (Figure 6, compare K,A; P,F; M,C; R,H; N,D; S,I). The same effects were also evident for BRAF clustering extent and PM density (Figure 6; green bars and curves in M,C; R,H; N,D; S,I). These results correlate with the relatively high aggressiveness of A375 cells and their associated morphological changes (Sriramarao and Bourdon, 1996).

Considering these changes between the A375 and 108T cell lines, we now turn to discuss the effect of vemurafenib on NRas and BRAF clustering in A375 cells. We observe that NRas and BRAF self-clustering, and their association in mutual clusters were significantly increased under vemurafenib treatment (Figure 6; compare P,K; Q,L; R,M; T,O, respectively). In contrast, BRAF densities at the PM were reduced under that treatment (yet non-significantly; Figure 6, compare N,S). This suggests that vemurafenib may have relatively complex and underappreciated (partly promoting, and partly inhibiting) effects on BRAF signaling, through its PM organization and interaction with NRas in mutual clusters. Indeed, RAF inhibitors have been shown to paradoxically activate the mitogenic pathway through wild-type RAF (Hatzivassiliou et al., 2010) and its dimers (Poulikakos et al., 2010).

### NRas is enriched at the periphery of Grb2 clusters

The mitogenic pathway involves the interaction of NRas with upstream proteins, downstream effectors, and regulating proteins. Since we found that NRas and BRAF organize in clusters at the PM, we turned to study NRas localization relative to related signaling molecules. Specifically, Grb2 is a small adapter protein that gets recruited to activated mitogenic receptors at the PM. It recruits SOS to the PM, which in turn acts as a RAS GEF (Giubellino et al., 2008).

**Figure 7:**
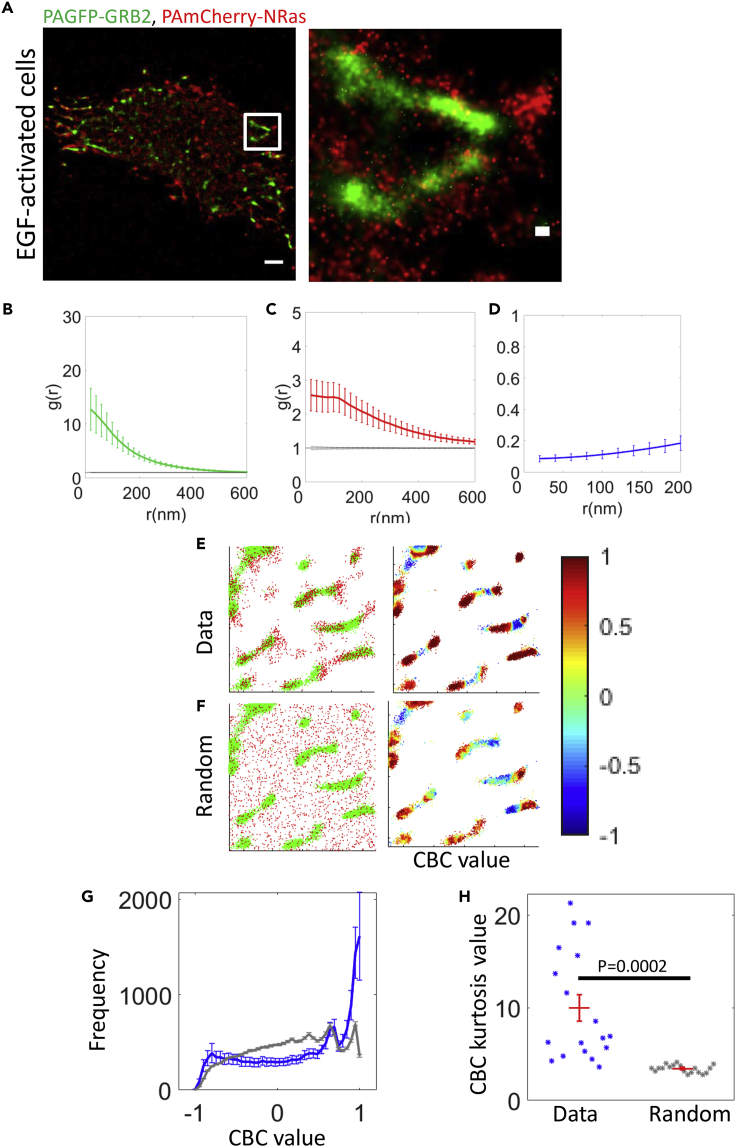
NRas localizes at the periphery of Grb2 clusters at the PM (A) Two-color PALM imaging of EGF-activated 108T melanoma cells expressing NRas-PAmCherry and PAGFP-GRB2. Cells were seeded on the coverslip for 2 days before fixation. Shown are representative cells (N = 22). Bars - 2 μm (left) and 200 nm (zoomed images). (B and C) PCF of PAGFP-GRB2 (B) and PAmCherry-NRas (C). (D) The EOM of NRas and GRB2 (averaged over all cells; see analyses for further details). (E) The CBC values color map (right) of Grb2 (green points in left image) relative to NRas (red points). [value of 1 in right image indicates the green points (Grb2) in the data that are close to red points (NRas)]. (F) The CBC values color map (right) of Grb2 (green points in left image) relative to randomly distribution red points (same number of red points from data, as in panel E). (G) The average frequency of CBC values of Grb2 relative to NRas for all cells for the data (blue) and for the randomly distribution red point (gray) (N = 22). (H) The kurtosis values of Grb2 CBC analysis for the data (blue) and the randomly distribution red points (gray).

We imaged PAGFP-Grb2 and PAmCherry-NRas in fixed cells by two-color PALM. Grb2 localized in pronounced clusters (Figures 7A and 7B). NRas molecules were more diffused at the membrane relative to Grb2 (Figure 7C). The EOM of NRas and Grb2 at short distances (<200 nm) had a relatively low value (<0.2), indicating an overall low interaction between NRas and Grb2 (Figure 7D). Nevertheless, we noted that NRas tends to localize at the periphery Grb2 clusters (Figure 7A, zoom and 6E, left).

To further study the relative organization of NRas and Grb2 at the PM, we turned to the complementary statistics of coordinate-based colocalization (CBC). Such statistics indicates the colocalization extent of two interacting proteins in each pixel of an image with values ranging between −1 (repulsion) to +1 (attraction) (Figure 7E). We compared the CBC scores of NRas and Grb2 to a model in which the location of Grb2 molecules was kept, while the location of NRas molecules was randomized across the studied field. This analysis showed that the CBC values were more extreme (i.e. closer to either +1 or −1) for the experimental data relative to the randomized fields. Points with CBC values of +1 were enriched at the edges of Grb2 clusters, while points with −1 values were enriched at the center of the clusters (compare Figures 7E and 7F). This relative enrichment in the experimental data relative to the random model could be clearly observed in the averaged histogram of CBC values for multiple cells (Figure 7G) and in the histogram kurtosis (Figure 7H).

To conclude, our two-color PALM images and statistical tests show that NRas is enriched at the periphery of Grb2 clusters at the PM. This indicates that NRas gets activated at the edges of such Grb2 clusters.

### NRas is enriched at the periphery of NF1 clusters

**Figure 8:**
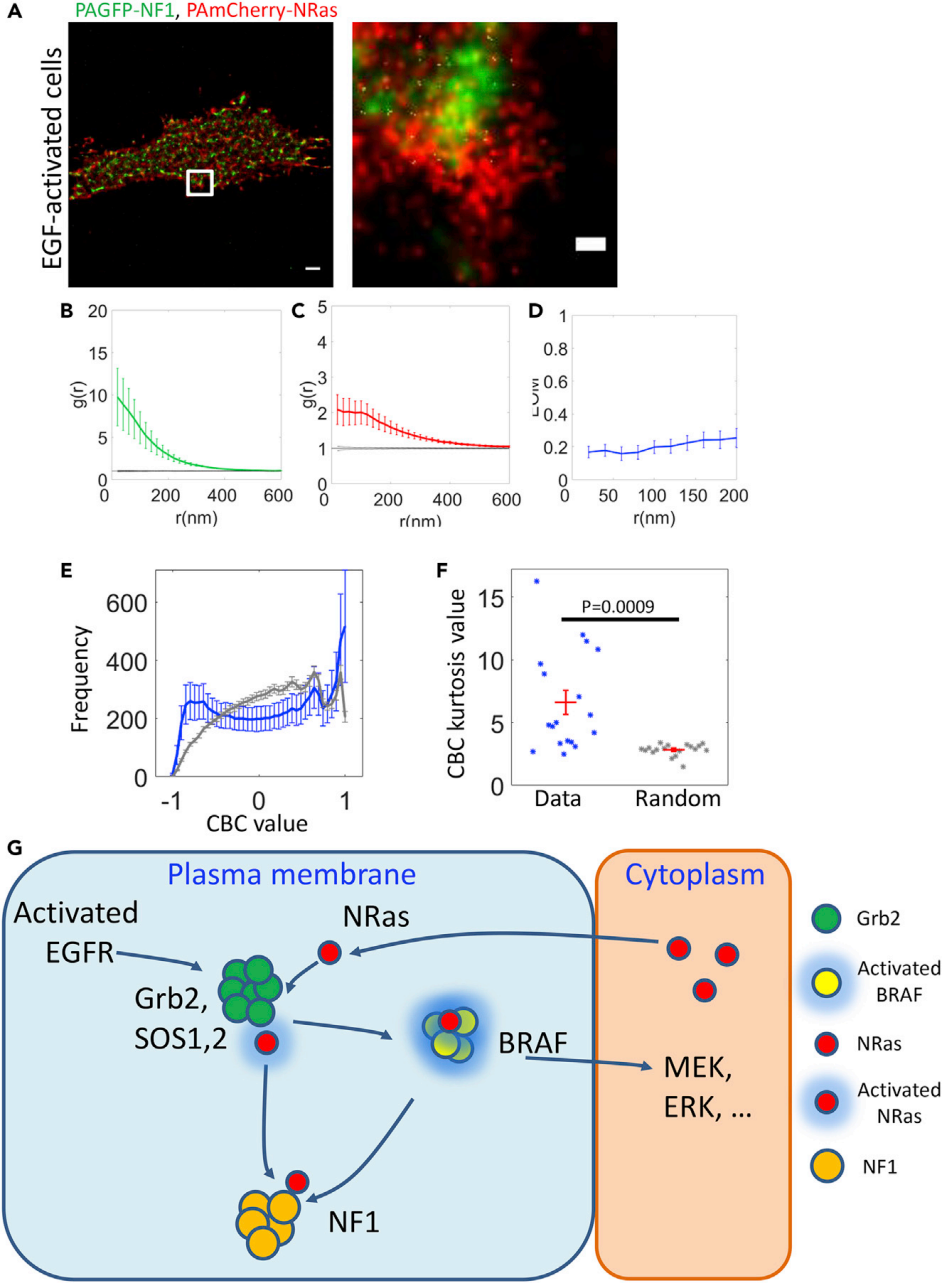
NRas localizes at the periphery of NF1 clusters and is regulated by signaling clusters at the PM (A) Two-color PALM imaging of EGF-activated 108T melanoma cells expressing NRas-PAmCherry and PAGFP-NF1. Cells were seeded on the coverslip for 2 days before fixation. Shown are representative cells (N = 21). Bars - 2 μm (left) and 200 nm (zoomed image). (B and C) PCF of PAGFP- NF1 and PAmCherry-NRas (red). (D) The EOM of NRas and NF1 (averaged over all cells; see analyses for further details). (E) The average frequency of CBC values of NF1 relative to NRas for all cells (blue) and the average frequency of CBC values of NF1 relative to randomly distribution red point (gray). (F) The kurtosis values of Grb2 CBC analysis for the data (blue) and for the randomly distribution red points (gray). (G) A schematic model of NRas activity regulation by signaling clusters at the PM.

We next studied whether this nanoscale organization and peripheral enrichment could occur for additional NRas-related signaling proteins at the PM. NF1 is a relatively large protein (2818 amino acids) that acts as an important negative regulator of Ras proteins (Marchuk et al., 1991), namely a RAS GAP. We visualized PAGFP-NF1 and PAmCherry-NRas in fixed cells by two-color PALM. Similar to Grb2, NF1 localized in pronounced clusters (Figures 8A, 8B, and 8C), and NRas molecules were relatively more dispersed at the PM (compare Figures 8B and 8C). The EOM of NRas and NF1 at short distances (<200 nm) also had a relatively low value (<0.2) (Figure 8D). Also, as for Grb2, NF1 seemed to preferentially localize at the periphery of NRas clusters (Figures 8A and S5). This peripheral localization was also found by the CBC statistics applied to multiple cells (N = 21; Figures S6B, 8E, and 8F).

Taken together, these results show a surprising nanoscale pattern in which dispersed NRas molecules associate with proximal, yet non-overlapping self-clusters of either Grb2 or NF1 at their periphery. These interactions serve to either activate NRas (by Grb2-SOS complexes) or deactivate NRas (by NF1). Such patterns could not be detected by diffraction-limited microscopy (Figure S5).

## Discussion

In this study, we applied two-color PALM to resolve the organization of individual NRas and associated signaling proteins in live melanoma cells, with resolution down to ∼20 nm. Specifically, we could resolve the coordinated and synchronized assembly (and disassembly) of NRas and BRAF in co-clusters at the PM of live, EGF-activated 108T melanoma cells. While SMLM imaging has a relatively limited temporal resolution, we could capture cluster dynamics, including formation, mobility, and integration with an effective time resolution of 5 s. We also found that NRas and BRAF co-localized with GPI domains, serving as possible nucleation sites, and used two drug inhibitors (lonafarnib and vemurafenib) to interfere with the self- and co-clustering of NRas and BRAF. Finally, we resolved the mutual organization of NRas and its related signaling proteins Grb2 and NF1. Surprisingly, NRas preferentially interacted with these regulating proteins at the periphery of self-clusters of these proteins.

Previously, KRas (Lee et al., 2019) and HRas (Prior et al., 2003) were studied using super-resolution imaging. While Ras isoforms (esp. NRas, KRas, and HRas) have similar sequence in their N-terminal (comprising effector and switches domains), they have major differences in the hypervariable region. This region is closely associated with the proteins organization and translocation at the PM (Hobbs et al., 2016). Our results show that mutual NRas and BRAF clusters are stable for ∼1–2 min. These results show a higher level of organization of NRas and of BRAF relative to the previous studies (Hu et al., 2013; Karnoub and Weinberg, 2008), which emphasized the dimerization of these proteins as isolated signaling units. In support of our findings, Mysore et al. have recently used combinations of molecular dynamics simulations, electron microscopy, and cell-based experiments to describe, in atomistic details, an intricate molecular assembly of mitogen-activated protein kinase-related proteins (Mysore et al., 2021). These structures included multiple (up to 8 or 16) active KRas molecules, having a composite interface with C-Raf, Galectin-3, and 14-3-3σ, which associated closely with and around this structure. Moreover, Mu et al. have recently developed an assay for inducing Ras clustering in live cells using nanobar patterns on the underlying coverslip (Mu et al., 2022). They observed relatively large Ras clusters that were visible using diffraction-limited microscopy, as they accumulated and were stabilized at the curved PM at the ends of the nanobars.

Our current results further extend our previous study, which showed NRas and BRAF nanoscale organization (Figure S1G) in fixed cells (Yakovian et al., 2021). Previous studies have often used truncated protein forms to study Ras-RAF interactions, e.g. the Ras-binding formation of RAF (e.g. (Mochizuki et al., 2001)). In contrast, we used here complete constructs (see STAR methods and Figure S1A). Labeling of these constructs was at the N-terminus of the proteins, which are furthest from the PM when the proteins are recruited to it. This served to avoid possible interruption of the tag with the recruitment and localization of the proteins at the PM.

Notably, SMLM imaging may carry additional complications. Most importantly, the proteins baring the fluorescent tags were overexpressed in the cells. Also, PALM imaging in live cells may restrict its ability to provide absolute counts of protein copy numbers. Thus, our results should be regarded as rough estimates of protein levels, or as relative between the different imaging conditions. Still, studying the relative organization of proteins using two-color PALM is less affected by such potential artifacts (Sherman et al., 2013).

Our observation of NRAs-BRAF co-clusters raises questions regarding their formation mechanisms and function in signaling. Previous studies have identified micro (nano)-domains as preferred sites for NRas activity (Prior et al., 2003). The mobility of Ras is hindered in such domains (Lee et al., 2019). We found here that both NRas and BRAF localized in PM domains enriched and highlighted by GPI. This indicates that such GPI-enriched domains might serve to nucleate clusters of NRas and BRAF that we have found in this study (van der Hoeven et al., 2013) (Zhou et al., 2015). Also, activity of NRas has been shown to correlate with its localization relative to nanometer-scale liquid-ordered domains (Shishina et al., 2018). This could account for the differences we observe in the fraction of NRas colocalized with the GPI domains between resting and EGF-activated cells.

Treatment of cancer cells with known drugs could shed further light on the underlying mechanisms for the association of NRas and BRAF in mutual clusters. The effects of the drugs seemed complex, as NRas and BRAF association in mutual clusters was reduced by the NRas farnesylation inhibitor lonafarnib, yet enhanced by the BRAF inhibitor vemurafenib. The lonafarnib results indicate that NRas farnesylation is needed for BRAF recruitment to NRas clusters and for intact BRAF signaling. In contrast, vemurafenib treatment seem to not only abrogate activity of oncogenic BRAF mutants but also decrease overall levels of BRAF at the PM. Unexpectedly, these fewer BRAF molecules now associate better with NRas clusters.

The propagation of mitogenic signals at the PM has not been extensively studied by super-resolution microscopy. We found here that NRas associates with self-clusters of the upstream protein Grb2, and of its key regulator NF1. NRas and BRAF clusters were localized at GPI-enriched clusters. Based on these findings, we propose a model in which the mitogenic signal (focusing on NRas) is propagated between relatively stationary and non-overlapping clusters at the PM, each conferring its own functional outcome on the signal: Grb2 clusters promote NRas activation (via SOS1,2); clustered BRAF gets activated by colocalization with activated NRas; and NF1 clusters downregulate NRas activity by their GTPase-enhancing activity (Figure 8G). Relatively dispersed NRas (in monomers or in small clusters) shuttle between these clusters to promote, mediate, and regulate its activity and signaling.

We hypothesize that our observed NRas-BRAF clusters and the NRas interactions with Grb2, NF1, and GPI in clusters could serve as novel targets for cancer therapy. For instance, our study sets the stage for finding the mediators of Ras and RAF co-clustering and its occurrence in additional Ras and RAF isoforms. It further sheds new light on the dynamics of NRas signaling, which is critical in health and disease.

### Limitations of the study

Our study relies on single-molecule localization microscopy. This method may suffer from multiple issues that could compromise its ability to provide absolute counts of molecules. While we address these issues in our image reconstruction and analyses, we recommend focusing the conclusions on relative changes in protein densities over time or under the various experimental conditions.

## STAR★Methods

### Key resources table

**Table:** 

REAGENT or RESOURCE	SOURCE	IDENTIFIER
**Chemicals, peptides, and recombinant proteins**		

Lipofetamin 3000	Invitrogen	L3000008
Vemorafenib	selleckchem	Cat#S1267
lonafarnib	Sigma-Aldrich	SML1457
EGF	Sigma-Aldrich	Cat#E9644

**Experimental models: Cell lines**		

108T	Surgery Branch of the National Cancer Institute	N/A
A375	Surgery Branch of the National Cancer Institute	N/A

**Recombinant DNA**		

Plasmid: PAmCherry-NRas	Yakovian et al.,2021	N/A
Plasmid: PAGFP-BRAF	Yakovian et al.,2021	N/A
Plasmid: GPI-PAmCherry	Yakovian et al.,2018	N/A
Plasmid: PAGF-NF1	This paper	N/A
Plasmid: PAGFP-Grb2	This paper	N/A

**Software and algorithms**		

ImageJ	Schneider et al., 2012	https://imagej.nih.gov/ij/
MATLAB	MATLAB ver. R2018b	https://www.mathworks.com/
ThunderSTORM	https://doi.org/10.1093/bioinformatics/btu202	https://zitmen.github.io/thunderstorm/

**Other**		

1.5 glass chambers coverslips	Ibidi	Cat#80827

### Resource availability

#### Lead contact

Further information and requests for resources and reagents should be directed to and will be fulfilled by the lead contact, Eilon Sherman (eilon.sherman@mail.huji.ac.il).

#### Materials availability

Plasmids generated in this study are available upon request.

### Experimental model and subject details

#### Cell lines

This study includes the patient-derived 108T melanoma cell-line (NRAS-Wild-Type, BRAF-Wild-Type and NF1-H1366Q). This cell line was used in a previous study (Alon et al., 2018) and derived from a panel of pathology-confirmed metastatic melanoma tumor resections collected from patient enrolled in Institutional Review Board (IRB)-approved clinical trials at the Surgery Branch of the National Cancer Institute (Bethesda, MD). Pathology-confirmed melanoma cell line was derived from mechanically or enzymatically dispersed tumor cells, which were then cultured in RPMI1640 + 10% FBS at 37C in 5% CO2 for 5–15 passages. The 108T cell line was tested negative for Mycoplasma.

### Method details

#### Samples

In this work, we constructed BRAF and NRas conjugated to the photoactivatable fluorophores PAGFP or PAmCherry, by the gateway cloning method (Katzen, 2007). BRAF and NRas constructs containing photoactivatable fluorescent proteins were cloned in pcDNA3 plasmids with a cytomegalovirus promoter. The cloning of the fluorescent tags was performed at the N-terminus to prevent localization disruption. Constructs were validated by DNA sequencing. The previously developed construct GPI-PAmCherry was already available for this work from our previous study (Yakovian et al., 2018). The 108T melanoma cells were transfected with two plasmids PAmCherry-NRas and PAGFP-BRAF or PAGFP-NRas and GPI-PAmCherry using Lipofetamin 3000 (L3000008, Invitrogen) for 48 h. Cell seeding and imaging was conducted on glass coverslips (#1.5 glass chambers, LabTek and Ibidi), and fixed with 2.4% paraformaldehyde for 30 min at 37C. For live imaging, 48 h post-transfection, cells were seeded on EGF coated coverslip and imaged.

#### Drug treatment

BRAFi Vemorafenib (Catalog No.S1267, Selleckchem) and the farnesyl transferase inhibitor lonafarnib (Sigma-Aldrich) were used in a concentration of 6 nM (Proietti et al., 2020) and 20 μM (Wang et al., 2017) respectively over 48 h.

#### Imaging

We conducted two-color PALM imaging similar to a previous study (Sherman et al., 2011) using a total internal reflection (TIRF) Nikon microscope with a CFI Apo TIRF X100 oil objective (NA 1.49, WD 0.12 mm). PAGFP and PAmCherry were photoactivated using variable intensity (0.5–10% of 30 mW in maximum) of 405 nm laser illumination that was changed gradually from beginning of imaging to the end and alternate excitation using (50% of 90 mW in maximum) 488 nm laser excitation for PAGFP and (50% of 90 mW in maximum) 561 nm for PAmCherry unchanged during the imaging. Laser illumination at all wavelengths covered a circular area with a diameter of 80 μm at the sample. Movies of fixed cell imaging were acquired for 2,000 frames at 13.1 fps and 12,000 frames at 50 fps for live cells, of an EMCCD Ixon^+^ camera. The focus of the microscope was maintained throughout the imaging using the PerfectFocus system of the microscope.

#### Analyses

We used the ThunderSTORM software (Ovesny et al., 2014), an ImageJ plugin (Schneider et al., 2012), to analyze PALM movies and generate images. Briefly, this software served to identify individual peaks and to assign them to individual molecules for rendering of the PALM images. The localization uncertainties of the different fluorophores used in our study peaked at approximately 20–30 nm (Figures S1B–S1F).

SMLM reconstruction requires grouping of individual peaks and their assignment to individual molecules. This process is performed over space and time, and so requires two threshold values of 'maximum off frame' and 'maximum grouping distance'. These values of grouping parameters were defined separately for each fluorophore in our previous study (Yakovian et al., 2021), following a detailed analysis of the fluorophores blinking statistics. A distance threshold and a temporal gap (Table S1) were employed for peak grouping to account for possible molecular blinking (Betzig et al., 2006). Temporal gaps were tested for each fluorophore separately to minimize possible over-counting of molecules as disc. The threshold values of 'maximum grouping distance' and the 'maximum off frames' were defined as shown in our previous study (Yakovian et al., 2021). These parameters were provided through dedicated filters during SMLM reconstruction using ThunderSTORM (Ovesny et al., 2014).

#### Clustering analyses of GPI and associated NRas molecules

In order to define GPI clusters and the boundary of their domains, we analysed the proteins localization through DBSCAN using the Matlab functions "rangesearch" (MathWorks) (Sander et al., 1998), as follows: First, our code defined and separated GPI proteins into groups of monomers, dimers, and clusters of 3, 4, or 5-mers. We set a distance of d_th_ = 80 nm as a threshold for defining molecules that belong to the same cluster (Figures S3A–S3F). This distance threshold corresponds to molecular density of GPI in micro domains. The cluster area and the number of NRas proteins in individual clusters were calculated for each GPI cluster separately. In this way, the density of NRas molecules within these domains and their density outside these domains were quantified. Over 20% of NRas proteins were populated in the GPI cluster domains, which covered less than 2% of the visualized area of the plasma membrane (i.e. the apparent cell footprint in our TIRF imaging). The sensitivity of this latter analysis was examined by choosing different distance threshold values of d_th_ = 60 nm and d_th_ = 100 nm (see Figures S3G and S3H and Table S2).

#### Second order statistics

A detailed description of the second-order statistics used in this study has been published and extensively described previously (Wiegand and Moloney, 2004). This description includes the univariate pair correlation function (PCF), the bivariate PCF (BPCF), and extent of mixing (EOM). Briefly, PCF [denoted here also as g(r)] describes and quantifies in a point pattern how density varies as a function of distance from a reference particle/point. Usually, PCF is normalized by the density of the sample, and was used in this normalized form throughout the study. The univariate PCF is used to explore a point pattern of a single species. It is further useful to compare the PCF results with a Poisson model that describes random placement of points across the field. This model results in a flat PCF, for which g(r) = 1. Thus, higher values of the PCF [i.e., g(r)] indicate self-clustering. Note that the notation g_11_(r) or g_22_(r) in Figure S1H relates to g(r) of either protein type 1 or 2, respectively.

For patterns with two species, BPCF statistics quantify the density of pattern 2 at distance r from an arbitrary point of pattern 1. To investigate whether or not two species (in a joint point pattern) are significantly interacting, we used the random labeling model. In this model, points of pattern 1 (n1) and points of pattern 2 (n2) distribute randomly at n1 + n2 fixed locations. Multiple Monte Carlo simulations replicate 19 times the point patterns while randomly relabeling the points (with the number of points from each species). The BPCF of the original point pattern g_12_(r) is then compared with the BPCFs of the simulations. We used the lowest and highest g_12_(r) of the different simulations as a 95% confidence interval for the acceptance or rejection of the model as a null hypothesis. Agreement of the data with the random labeling (RL) model indicates homogeneous mixing, and hence strong interaction (in a statistical sense) of the two species under study. Alternatively, a model of no interaction would result in a flat curve where g_12_(r) = 1. The EOM represents the average of BPCF over multiple cells using 95% confidence interval due to the RL model. EOM is normalized between the values of +1 for perfect mixing and 0 for the model of no interaction. All analyses for a single co-cluster were performed on an area of 1.5 × 1.5 μm.

#### Coordinate-based colocalization analysis

To quantify the colocalization of two species (e.g. two types of proteins - A and B) in a joint point pattern data, we used Coordinate-based colocalization (CBC) analysis. Using this method we defined for each point in pattern A the level of co-localization with regard to another points pattern B. The CBC value for each A protein is determined according to the number of B proteins around this A point as the function of distance. All calculations were based on the article by Malkusch et al. (Malkusch et al., 2012). The CBC values can range from −1 to +1, indicating anti-colocalization and perfect colocalization, respectively (Figure S6A). For simulation, the same number of NRas proteins from the experimental data, were randomly spread across the field 19 times and CBC values were calculated for each cell. To define the CBC values for each point, the rank correlation coefficient (Spearman), were calculated for 50 rings with a radius varying from 20 nm to 1000 nm around the reference point. The radial jumps of 20 nm were chosen according to the localization uncertainty of two color PALM of ∼20 nm. The maximal radius was chosen according to the existence of cluster structures with the size of a few hundred nanometres (as found by PCF analyses for the imaged proteins in this study).

### Quantification and statistical analysis

For the statistical analysis of the protein density or EOM data are judged to be statistically significant when p < 0.05 by two-tailed Student’s *t* test. The number of cells is indicated in each Figure legends.

## Data Availability

All data produced in this study are included in the published article and its supplementary information, or are available from the lead contact upon request. This paper does not report original code. Any additional information required to reanalyze the data reported in this paper is available from the lead contact upon request.

## References

[bib1] Alon M., Arafeh R., Lee J.S., Madan S., Kalaora S., Nagler A., Abgarian T., Greenberg P., Ruppin E., Samuels Y. (2018). CAPN1 is a novel binding partner and regulator of the tumor suppressor NF1 in melanoma. Oncotarget.

[bib2] Arafeh R., Qutob N., Emmanuel R., Keren-Paz A., Madore J., Elkahloun A., Wilmott J.S., Gartner J.J., Di Pizio A., Winograd-Katz S. (2015). Recurrent inactivating RASA2 mutations in melanoma. Nat. Genet..

[bib3] Betzig E., Patterson G.H., Sougrat R., Lindwasser O.W., Olenych S., Bonifacino J.S., Davidson M.W., Lippincott-Schwartz J., Hess H.F. (2006). Imaging intracellular fluorescent proteins at nanometer resolution. Science.

[bib4] Bollag G., Clapp D.W., Shih S., Adler F., Zhang Y.Y., Thompson P., Lange B.J., Freedman M.H., Mccormick F., Jacks T., Shannon K. (1996). Loss of NF1 results in activation of the Ras signaling pathway and leads to aberrant growth in haematopoietic cells. Nat. Genet..

[bib5] Brooks S. (1998). Markov chain Monte Carlo method and its application. J. Roy. Stat. Soc. D.

[bib6] Cargnello M., Roux P.P. (2012). Activation and function of the MAPKs and their substrates, the MAPK-activated protein kinases. Microbiol. Mol. Biol. Rev..

[bib7] Eisenberg S., Beckett A.J., Prior I.A., Dekker F.J., Hedberg C., Waldmann H., Ehrlich M., Henis Y.I. (2011). Raft protein clustering alters N-Ras membrane interactions and activation pattern. Mol. Cell Biol..

[bib8] Giubellino A., Burke T.R., Bottaro D.P. (2008). Grb2 signaling in cell motility and cancer. Expert Opin. Ther. Targets.

[bib9] Griffiths G., Lucocq J.M. (2014). Antibodies for immunolabeling by light and electron microscopy: not for the faint hearted. Histochem. Cell Biol..

[bib10] Hatzivassiliou G., Song K., Yen I., Brandhuber B.J., Anderson D.J., Alvarado R., Ludlam M.J.C., Stokoe D., Gloor S.L., Vigers G. (2010). RAF inhibitors prime wild-type RAF to activate the MAPK pathway and enhance growth. Nature.

[bib11] Hobbs G.A., Der C.J., Rossman K.L. (2016). RAS isoforms and mutations in cancer at a glance. J. Cell Sci..

[bib12] Hu J., Stites E.C., Yu H., Germino E.A., Meharena H.S., Stork P.J.S., Kornev A.P., Taylor S.S., Shaw A.S. (2013). Allosteric activation of functionally asymmetric RAF kinase dimers. Cell.

[bib13] Karnoub A.E., Weinberg R.A. (2008). Ras oncogenes: split personalities. Nat. Rev. Mol. Cell Biol..

[bib14] Katzen F. (2007). Gateway((R)) recombinational cloning: a biological operating system. Expet Opin. Drug Discov..

[bib15] Keshet Y., Seger R. (2010). The MAP kinase signaling cascades: a system of hundreds of components regulates a diverse array of physiological functions. Methods Mol. Biol..

[bib16] Khan I., Rhett J.M., O'bryan J.P. (2020).

[bib17] Lee Y., Phelps C., Huang T., Mostofian B., Wu L., Zhang Y., Tao K., Chang Y.H., Stork P.J., Gray J.W. (2019). High-throughput, single-particle tracking reveals nested membrane domains that dictate KRas(G12D) diffusion and trafficking. Elife.

[bib18] Leung Y.H., Guo M.Y., Ma A.P.Y., Ng A.M.C., Djurišić A.B., Degger N., Leung F.C.C. (2017). Transmission electron microscopy artifacts in characterization of the nanomaterial-cell interactions. Appl. Microbiol. Biotechnol..

[bib19] Malkusch S., Endesfelder U., Mondry J., Gelléri M., Verveer P.J., Heilemann M. (2012). Coordinate-based colocalization analysis of single-molecule localization microscopy data. Histochem. Cell Biol..

[bib20] Marchuk D.A., Saulino A.M., Tavakkol R., Swaroop M., Wallace M.R., Andersen L.B., Mitchell A.L., Gutmann D.H., Boguski M., Collins F.S. (1991). Cdna cloning of the type-1 neurofibromatosis gene - complete sequence of the Nf1 gene-product. Genomics.

[bib21] Mochizuki N., Yamashita S., Kurokawa K., Ohba Y., Nagai T., Miyawaki A., Matsuda M. (2001). Spatio-temporal images of growth-factor-induced activation of Ras and Rap1. Nature.

[bib22] Morrison D.K. (2012). MAP kinase pathways. Cold Spring Harbor Perspect. Biol..

[bib23] Mu H., Zeng Y., Zhuang Y., Gao W., Zhou Y., Rajalingam K., Zhao W. (2022). Patterning of oncogenic Ras clustering in live cells using vertically aligned nanostructure arrays. Nano Lett..

[bib24] Mysore V.P., Zhou Z.W., Ambrogio C., Li L., Kapp J.N., Lu C., Wang Q., Tucker M.R., Okoro J.J., Nagy-Davidescu G. (2021). A structural model of a Ras-Raf signalosome. Nat. Struct. Mol. Biol..

[bib25] Nan X., Tamgüney T.M., Collisson E.A., Lin L.J., Pitt C., Galeas J., Lewis S., Gray J.W., Mccormick F., Chu S. (2015). Ras-GTP dimers activate the mitogen-activated protein kinase (MAPK) pathway. Proc. Natl. Acad. Sci. USA.

[bib26] Ovesný M., Křížek P., Borkovec J., Svindrych Z., Hagen G.M. (2014). ThunderSTORM: a comprehensive ImageJ plug-in for PALM and STORM data analysis and super-resolution imaging. Bioinformatics.

[bib27] Parkkola H., Siddiqui F.A., Oetken-Lindholm C., Abankwa D. (2021). FLIM-FRET analysis of Ras nanoclustering and membrane-anchorage. Methods Mol. Biol..

[bib28] Poulikakos P.I., Zhang C., Bollag G., Shokat K.M., Rosen N. (2010). RAF inhibitors transactivate RAF dimers and ERK signalling in cells with wild-type BRAF. Nature.

[bib29] Prior I.A., Muncke C., Parton R.G., Hancock J.F. (2003). Direct visualization of Ras proteins in spatially distinct cell surface microdomains. J. Cell Biol..

[bib30] Proietti I., Skroza N., Michelini S., Mambrin A., Balduzzi V., Bernardini N., Marchesiello A., Tolino E., Volpe S., Maddalena P. (2020). BRAF inhibitors: molecular targeting and immunomodulatory actions. Cancers.

[bib31] Samatar A.A., Poulikakos P.I. (2014). Targeting RAS-ERK signalling in cancer: promises and challenges. Nat. Rev. Drug Discov..

[bib32] Sander J., Ester M., Kriegel H.P., Xu X. (1998). Density-based clustering in spatial databases: the algorithm GDBSCAN and its applications. Data Min. Knowl. Discov..

[bib33] Santarpia L., Lippman S.M., El-Naggar A.K. (2012). Targeting the MAPK-RAS-RAF signaling pathway in cancer therapy. Expert Opin. Ther. Targets.

[bib34] Schlessinger J. (1993). How receptor tyrosine kinases activate Ras. Trends Biochem. Sci..

[bib35] Schmick M., Kraemer A., Bastiaens P.I.H. (2015). Ras moves to stay in place. Trends Cell Biol..

[bib36] Schneider C.A., Rasband W.S., Eliceiri K.W. (2012). NIH Image to ImageJ: 25 years of image analysis. Nat. Methods.

[bib37] Schubbert S., Shannon K., Bollag G. (2007). Hyperactive Ras in developmental disorders and cancer. Nat. Rev. Cancer.

[bib38] Sharma P., Varma R., Sarasij R.C., Ira, Gousset K., Gousset K., Krishnamoorthy G., Rao M., Mayor S. (2004). Nanoscale organization of multiple GPI-anchored proteins in living cell membranes. Cell.

[bib39] Sherman E., Barr V., Manley S., Patterson G., Balagopalan L., Akpan I., Regan C.K., Merrill R.K., Sommers C.L., Lippincott-Schwartz J., Samelson L.E. (2011). Functional nanoscale organization of signaling molecules downstream of the T cell antigen receptor. Immunity.

[bib40] Sherman E., Barr V.A., Samelson L.E. (2013). Resolving multi-molecular protein interactions by photoactivated localization microscopy. Methods.

[bib41] Shishina A.K., Kovrigina E.A., Galiakhmetov A.R., Rathore R., Kovrigin E.L. (2018). Study of forster resonance energy transfer to lipid domain markers ascertains partitioning of semisynthetic lipidated N-Ras in lipid raft nanodomains. Biochemistry.

[bib42] Simons K., Ikonen E. (1997). Functional rafts in cell membranes. Nature.

[bib43] Sriramarao P., Bourdon M.A. (1996). Melanoma cell invasive and metastatic potential correlates with endothelial cell reorganization and tenascin expression. Endothelium.

[bib44] Subach F.V., Patterson G.H., Manley S., Gillette J.M., Lippincott-Schwartz J., Verkhusha V.V. (2009). Photoactivatable mCherry for high-resolution two-color fluorescence microscopy. Nat. Methods.

[bib45] Terrell E.M., Morrison D.K. (2019). Ras-mediated activation of the Raf family kinases. Cold Spring Harb. Perspect. Med..

[bib46] van der Hoeven D., Cho K.J., Ma X., Chigurupati S., Parton R.G., Hancock J.F. (2013). Fendiline inhibits K-Ras plasma membrane localization and blocks K-Ras signal transmission. Mol. Cell Biol..

[bib47] Wang J., Lian Y., Gu Y., Wang H., Gu L., Huang Y., Zhou L., Huang Y. (2017). Synergistic effect of farnesyl transferase inhibitor lonafarnib combined with chemotherapeutic agents against the growth of hepatocellular carcinoma cells. Oncotarget.

[bib48] Wiegand T., Moloney K. (2004). Rings, circles, and null-models for point pattern analysis in ecology. Oikos.

[bib49] Yakovian O., Sajman J., Arafeh R., Neve-Oz Y., Alon M., Samuels Y., Sherman E. (2021). MEK inhibition reverses aberrant signaling in melanoma cells through reorganization of NRas and BRAF in self nano-clusters. Cancer Res..

[bib50] Yakovian O., Schwarzer R., Sajman J., Neve-Oz Y., Razvag Y., Herrmann A., Sherman E. (2018). Gp41 dynamically interacts with the TCR in the immune synapse and promotes early T cell activation. Sci. Rep..

[bib51] Zhou Y., Hancock J.F. (2015). Ras nanoclusters: versatile lipid-based signaling platforms. Biochim. Biophys. Acta.

[bib52] Zhou Y., Prakash P., Liang H., Cho K.J., Gorfe A.A., Hancock J.F. (2017). Lipid-sorting specificity encoded in K-Ras membrane anchor regulates signal output. Cell.

[bib53] Zhou Y., Wong C.O., Cho K.J., Van Der Hoeven D., Liang H., Thakur D.P., Luo J., Babic M., Zinsmaier K.E., Zhu M.X. (2015). SIGNAL TRANSDUCTION. Membrane potential modulates plasma membrane phospholipid dynamics and K-Ras signaling. Science.

