# Supplementary material for: NRas activity is regulated by dynamic interactions with nanoscale signaling clusters at the plasma membrane
Source: iScience. 2022 Oct 9;25(11):105282. doi: 10.1016/j.isci.2022.105282 (PMC9593252; doi:10.1016/j.isci.2022.105282)
Supplement: Document S1. Figures S1–S6 and Tables S1 and S2 [file mmc1.pdf]

iScience, Volume *25*

Supplemental information

NRas activity is regulated by dynamic interactions with nanoscale signaling clusters at the plasma membrane

Oren Yakovian, Julia Sajman, Michal Alon, Rand Arafeh, Yardena Samuels, and Eilon Sherman

Supplemental Information

NRas activity is regulated by dynamic interactions with nanoscale signaling clusters at the plasma membrane

Oren Yakovian[1], Julia Sajman[1], Michal Alon[2], Rand Arafeh[2,3], Yardena Samuels[2], Eilon Sherman[1,4,*]

[1]*Racah Institute of Physics, The Hebrew University, Jerusalem, Israel, 91904*
[2]*Department of Molecular Cell Biology, Weizmann Institute of Science, 7610001 Rehovot, Israel*
[3]*Department of Molecular Oncology, Dana Farber Cancer Institute, Boston, MA, USA*
[4]*Lead contact*
* - correspondence: eilon.sherman@mail.huji.ac.il

Supplemental Tables

Parameter \ Fluorophore	PAGFP	PAmCherry
Maximum off frames	15	25
Maximum distance (nm)	75	100

Table S1. The grouping threshold values of PAGFP and PAmCherry, Related to Figures 1-8.

	Distance threshold \ Parameter	The fraction of NRas proteins in GPI domain*	The fraction of PM area covered by GPI domains*
Resting cells	60 nm	13.8 ± 3.3 %	0.64 ± 0.32 %
	80 nm	21.3 ± 3.7 %	1.31 ± 0.51 %
	100 nm	27.3 ± 4.0 %	2.26 ± 0.74 %
EGF-activated cells	60 nm	7.6 ± 1.8 %	0.44 ± 0.10 %
	80 nm	16.1 ± 2.9 %	1.51 ± 0.34 %
	100 nm	18.0 ± 3.0 %	1.98 ± 0.37 %

- \- Errors are SEM

Table S2. The sensitivity of GPI cluster analysis to the distance threshold, Related to Figures 4,5.

Supplemental Figures and Legends

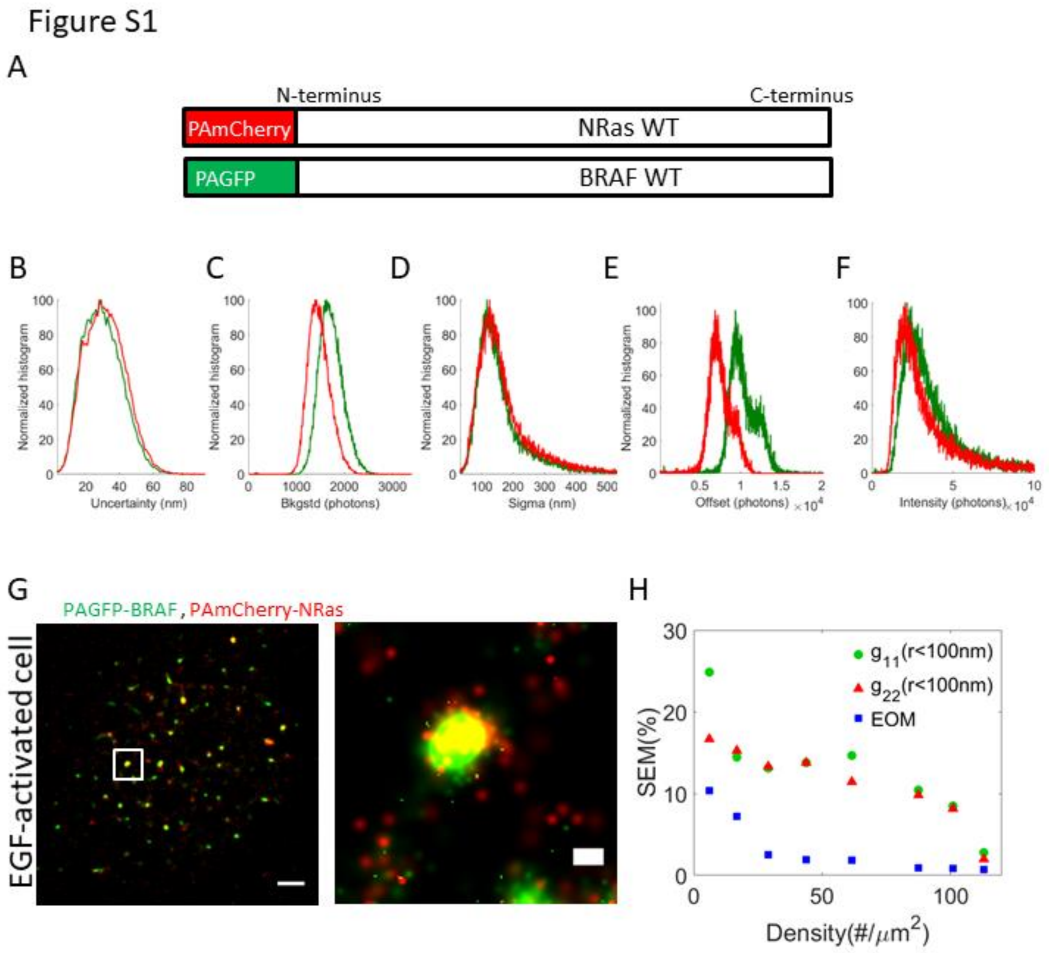

Figure S1. Two-color PALM imaging of fluorescently-tagged NRas and BRAF, Related to Figures 1-8.

(A) PAGFP and PAmCherry fluorescent tags were cloned at the N-terminus of wild type NRas or BRAF to prevent localization disruption. (B-F) Histograms of localization parameters for PAGFP-BRAF and PAmCherry-NRas, as imaged by two-color PALM. Parameters include: localization uncertainties, Background standard deviation (std), Sigma (of the Gaussian fit), Offset and Intensity. Parameter values were provided by

ThunderSTORM (See Methods for further details). (G) Two-color PALM imaging of 108T melanoma cells expressing PAmCherry-NRas and PAGFP-BRAF. Cells were seeded on the coverslip for 2 days and activated with FGF before fixation. (H) Fluctuations in the measures of g_{11}(r<100nm), g_{22}(r<100nm), and EOM as a function of molecular density in individual clusters under study. Bars, 2 µm (left) and 200 nm (right).

Figure S2

NRas, BRAF

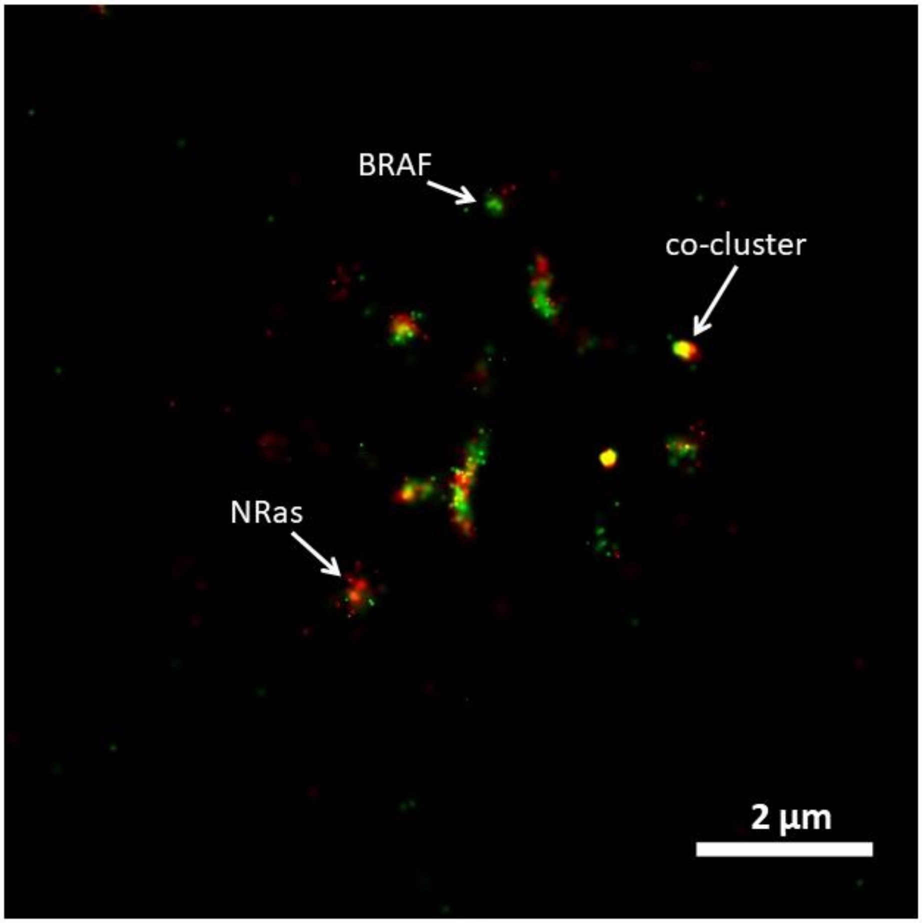

Figure S2. Co-clustering dynamics of NRas and BRAF, Related to Figure 1.

A snapshot from a movie (M1) of PM recruitment and co-cluster formation (in regions 1,2,3) or disintegration (in regions 1,2). The movie was captured using two-color PALM live imaging of NRas and BRAF in 108T melanoma cell.

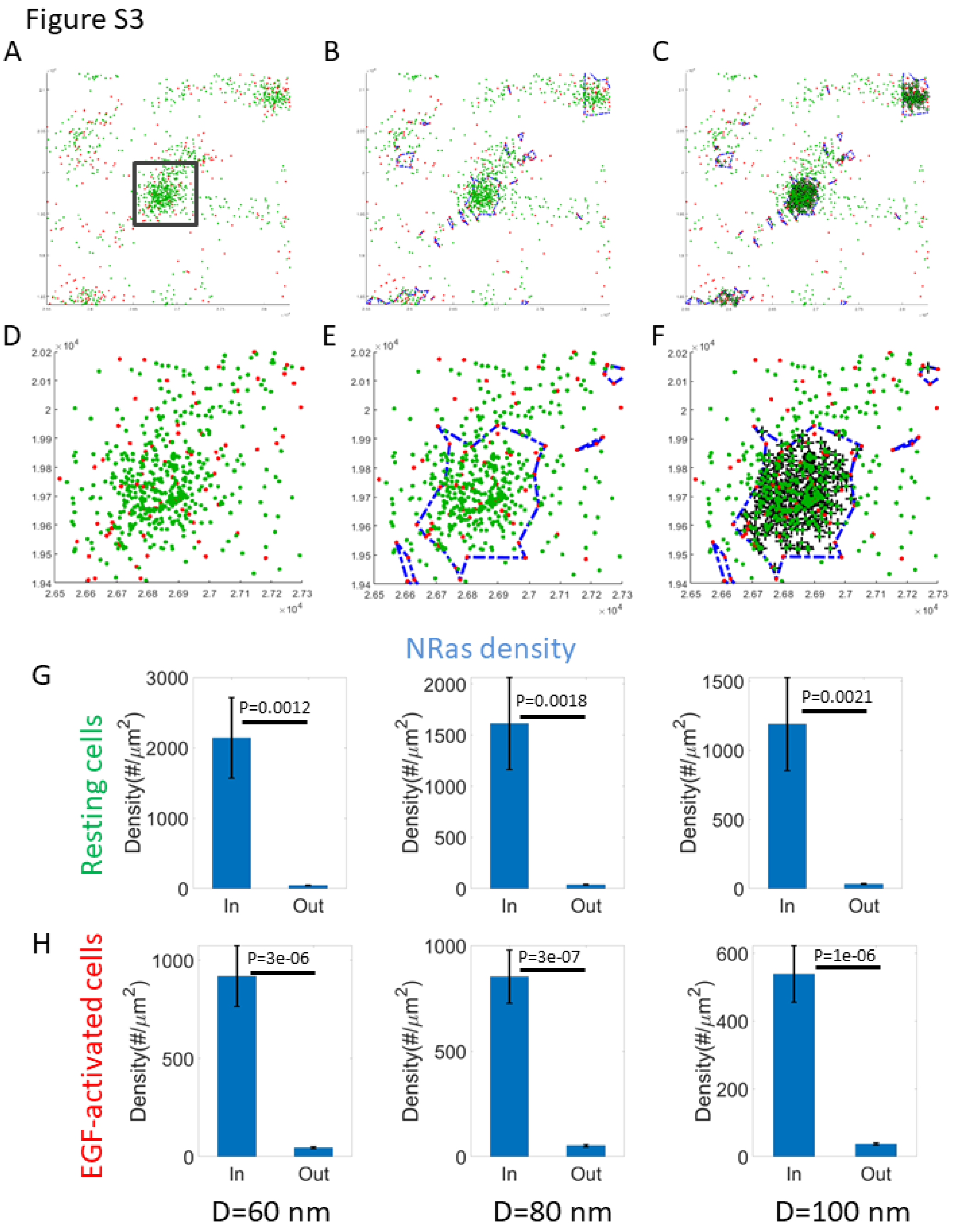

Figure S3. Cluster analyses of NRas and GPI organization, Related to Figures 4,5.

(A, D) Two-color PALM imaging of GPI-PAmCherry (red points) and PAGFP-NRas (green points) in a fixed melanoma cell. D shows the zoom region (black rectangle) in A.

(B, E) Selected GPI-enriched region (blue polygons) in representative area of NRas and GPI at the PM. Red points (GPI) are defined as a cluster according to their proximity from other red points. (C, F) Green points (NRas) are defined to be either in or out of GPI-enriched domains (see Methods). NRas molecules in clusters are highlighted using black crosses. NRas outside clusters is not further highlighted, and shows as green dots. (G, H) The density of NRas in and out of the GPI-enriched domains at the PM of the resting and EGF-activated melanoma cells using different distance thresholds for cluster definition. In order to examine the sensitivity of this analysis to the chosen distance threshold, we set threshold distances of 60, 80, and 100 nm. p-values were calculated using student's t-test.

Figure S4

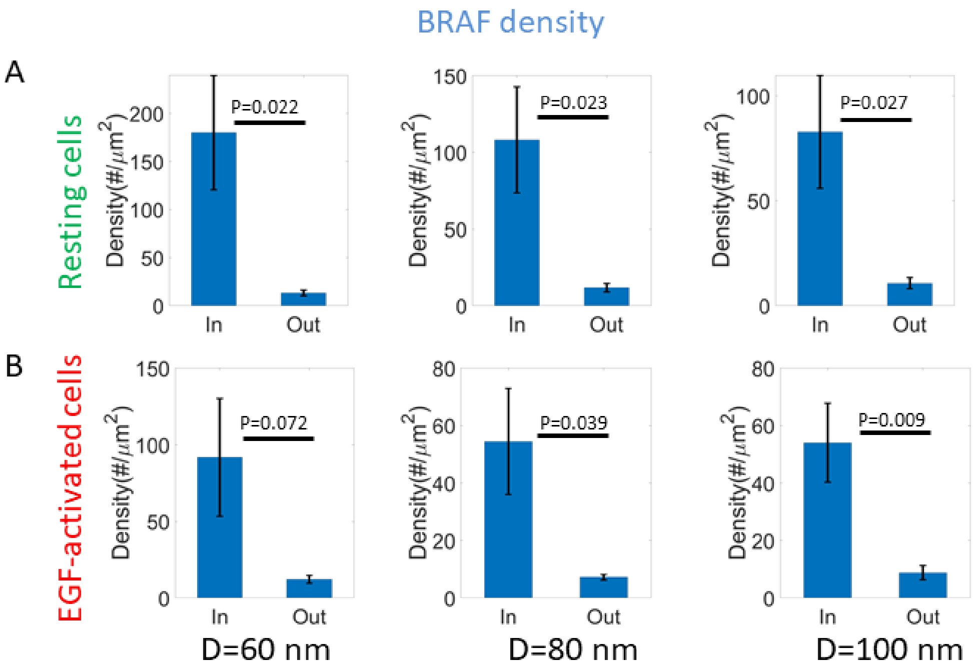

Figure S4. Cluster analyses of BRAF and GPI organization, Related to Figures 4,5.

(A, B) The density of BRAF in and out of the GPI-enriched domains at the PM of the (A) resting and (B) EGF-activated melanoma cells using different distance thresholds for cluster definition. In order to examine the sensitivity of this analysis to the chosen distance threshold, we set threshold distances of 60, 80, and 100 nm. p-values were calculated using student's t-test.

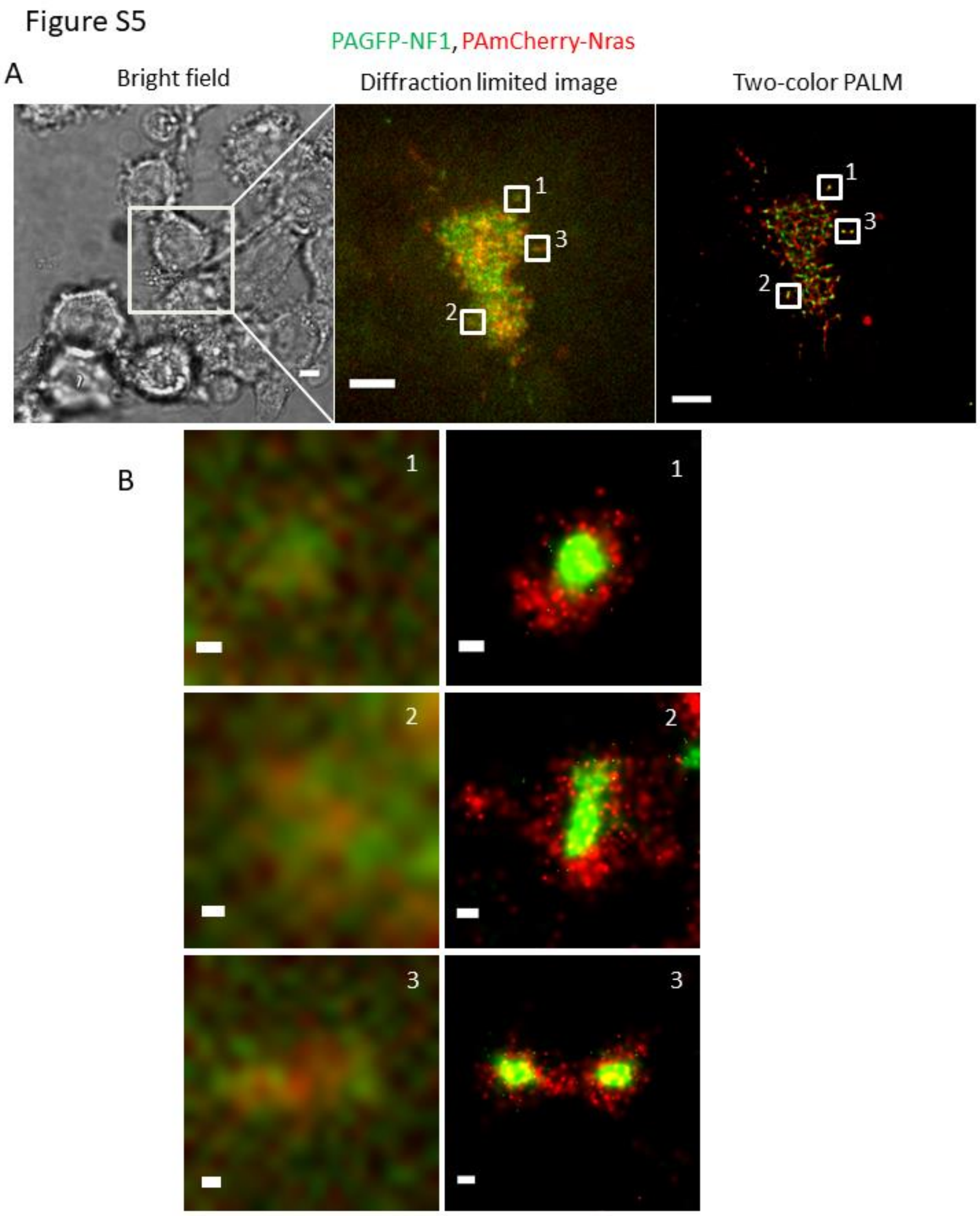

Figure S5. The organization of NRas and NF1 clusters at the PM, Related to Figure 8.

(A) Bright field, fluorescent, and two-color PALM image of a melanoma cell expressing PAGF-NF1 (green) and PAmCherry-NRas (red). Bars - 5µm. (B) The localization of NRas

proteins at the periphery of NF1 clusters are shown in selected zoom regions, and can be distinguished only by super resolved images using two-color PALM. Bars – 200 nm.

Figure S6

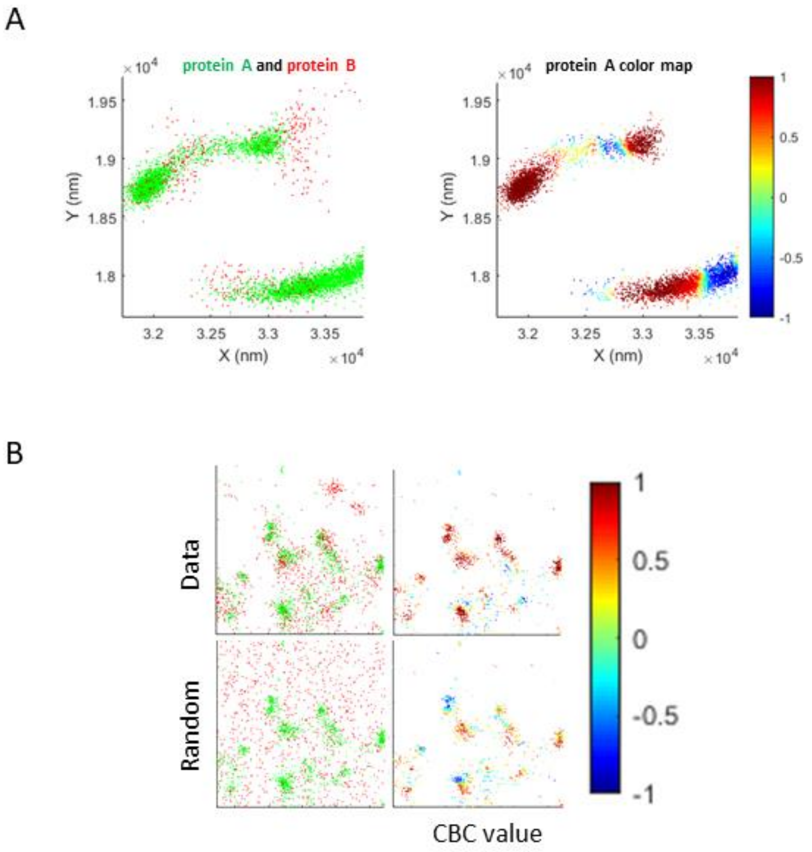

Figure S6. The CBC color-map and randomly simulations, Related to Figure 8.

(A) The CBC values color map (right) of protein A (green points in left image) relative to protein B (red points). [value of 1 in right image indicates the green points in the data that are close to red points]. (B) The CBC values color map (right) of NF1 (green points in left image) relative to NRas (red points) in the top row, and randomly distributed of red point (same number of NRas proteins in data) on the field of NF1 clusters in the bottom.
